# Metal–Organic Frameworks/Heterojunction Structures for Surface-Enhanced Raman Scattering with Enhanced Sensitivity and Tailorability

**DOI:** 10.1021/acsami.4c01588

**Published:** 2024-05-08

**Authors:** Wenwen Yuan, Keran Jiao, Hang Yuan, Hongzhao Sun, Eng Gee Lim, Ivona Mitrovic, Sixuan Duan, Shan Cong, Ruiqi Yong, Feifan Li, Pengfei Song

**Affiliations:** † School of Advanced Technology, Xi’an Jiaotong - Liverpool University, Suzhou 215123, China; ‡ Department of Electrical Engineering and Electronics, University of Liverpool, Liverpool L69 7ZX, U.K.; § State Key Laboratory for Manufacturing Systems Engineering, Xi’an Jiaotong University, Xi’an 710049, China; ∥ School of Physical Science and Technology, Suzhou University of Science and Technology, Suzhou 215009, China; ⊥ Key Laboratory of Bionic Engineering, Jilin University, Changchun 130022, China; # School of Nano-Tech and Nano-Bionics, University of Science and Technology of China, Suzhou 215123, China

**Keywords:** surface-enhanced Raman scattering, metal−organic frameworks, heterojunction, efficient photoinduced charge transfer

## Abstract

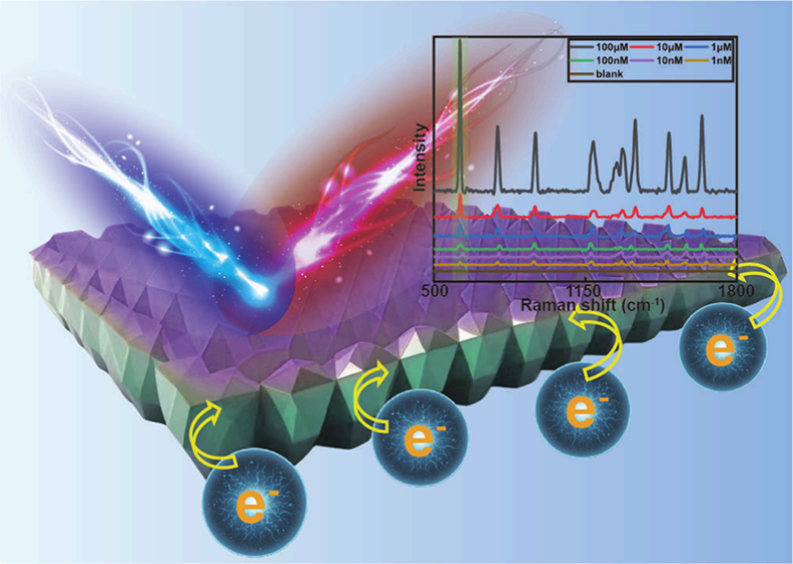

Metal–organic frameworks (MOFs), which are composed of crystalline microporous materials with metal ions, have gained considerable interest as promising substrate materials for surface-enhanced Raman scattering (SERS) detection via charge transfer. Research on MOF-based SERS substrates has advanced rapidly because of the MOFs’ excellent structural tunability, functionalizable pore interiors, and ultrahigh surface-to-volume ratios. Compared with traditional noble metal SERS plasmons, MOFs exhibit better biocompatibility, ease of operation, and tailorability. However, MOFs cannot produce a sufficient limit of detection (LOD) for ultrasensitive detection, and therefore, developing an ultrasensitive MOF-based SERS substrate is imperative. To the best of our knowledge, this is the first study to develop an MOFs/heterojunction structure as an SERS enhancing material. We report an *in situ* ZIF-67/Co(OH)_2_ heterojunction-based nanocellulose paper (nanopaper) plate (*in situ* ZIF-67 nanoplate) as a device with an LOD of 0.98 nmol/L for Rhodamine 6G and a Raman enhancement of 1.43 × 10^7^, which is 100 times better than that of the pure ZIF-67-based SERS substrate. Further, we extend this structure to other types of MOFs and develop an *in situ* HKUST-1 nanoplate (with HKUST-1/Cu(OH)_2_). In addition, we demonstrate that the formation of heterojunctions facilitates efficient photoinduced charge transfer for SERS detection by applying the M_*x*_(OH)_*y*_-assisted (where M = Co, Cu, or other metals) MOFs/heterojunction structure. Finally, we successfully demonstrate the application of medicine screening on our nanoplates, specifically for omeprazole. The nanoplates we developed still maintain the tailorability of MOFs and perform high anti-interference ability. Our approach provides customizing options for MOF-based SERS detection, catering to diverse possibilities in future research and applications.

## Introduction

Metal–organic frameworks (MOFs) are novel classes of supramolecular materials that combine mixed organic–inorganic semiconductor properties and are composed of metal nodes and organic connectors.^1−4^ Recently, MOFs have emerged as promising materials in many fields, including chemistry, physics, materials science, engineering, and biology, because of their highly porous features and excellent structural tunability.^5−8^ Therefore, expanding the potential range of their use beyond traditional applications is crucial for exploiting their new properties. Surface-enhanced Raman scattering (SERS), which is a potent trace analytical method for characterizing nanostructures on the chemical structure and composition of molecules with high sensitivity, is an attractive application area of MOF study recently.^9−12^ SERS substrate materials are critical for SERS detection to enhance SERS signals.^13,14^

MOFs have been extensively studied in SERS over the past decade owing to their advantages, such as low cost, high chemical stability, inherent structural flexibility, and greater exchangeability of metal centers compared to that of traditional metal SERS plasmons (noble metals).^15−18^ Cong et al. achieved a major breakthrough by identifying the SERS enhancement mechanism of MOFs.^9^ They found that, like other semiconductors, MOFs’ Raman enhancement is provided by the existence of charge transfer (CT) at the semiconductor–analyte contact area.^6^ The distinctive properties of MOFs render them appealing as materials for SERS detection in several ways. For example, the structural flexibility of MOFs enables the optimization of CT interactions between the SERS substrate and probe molecules, thereby enhancing the signal intensity.^19^ Further, the ultrahigh specific surface area-to-volume ratio and functionalized pore interior of MOFs can help enrich probe molecules.^10^ Although MOFs are promising for SERS detection because of the above advantages, they result in a very high limit of detection (LOD) because of prohibiting analytes and the small surface micropores of MOFs, which fails to satisfy the actual ultrasensitive detection requirements.^9,20^ Therefore, developing an ultrasensitive and easy-to-operate MOF-based SERS substrate is essential.

Thus far, three methods have been reported for increasing the MOFs-based SERS sensitivity. One method is to establish plasmonic nanoparticle (PNP)–MOF bicomponent hybrid systems. Target molecules’ SERS signals can be amplified by PNPs through electromagnetic mechanism (EM) enhancement, and MOFs provide additional less effective SERS enhancement by a chemical mechanism (CM).^21,22^ Another method involves fabricating a multicomponent MOF structure by generating an MOF-supported internoble metal layer. However, these two methods cannot utilize the advantages of selectivity and flexibility of MOFs, and MOFs can only be used to increase the sensitivity of the traditional metal plasmon.^23^ Moreover, these methods increase the SERS sensitivity of pure MOFs materials by only 10-fold. The third method that improves the sensitivity of MOFs themselves is to develop photoinduced oxygen vacancy defects in MOF materials to effectively reduce the LOD of the compound.^24^ However, the photoexcitation of the MOF-based SERS substrate remained stable only for 2 weeks and could not be utilized as a steady-state method. In addition, all of these methods require complicated fabrication processes. To the best of our knowledge, a stable and ultrasensitive MOFs-based SERS substrate that can be fabricated by a facile process has not yet been developed.

In this study, for the first time, we realized MOFs/heterojunction (M_*x*_(OH)_*y*_-assisted, M = Co, Cu, or other metals) as SERS substrates. The scattering cross sections are known to grow due to the photoinduced charge transfer (PICT) between the semiconductor and adsorbed molecules, hence amplifying the Raman signals.^25^ As a proof of concept, we first developed an *in situ* ZIF-67/Co(OH)_2_ heterojunction-based nanocellulose paper (nanopaper) plate (*in situ* ZIF-67 nanoplate) for use as an SERS detection device. The developed nanopaper has an ultrasmooth surface, high optical transparency, and tunable chemical properties, which is an excellent SERS substrate material.^26−31^ Moreover, it is an excellent platform for the facile generation of MOF/heterojunction structures through a simple ion layer absorption and reaction process.^32−36^ The ZIF-67 nanoplate achieved an SERS LOD of 10^–10^ mol/L (0.98 nmol/L) using Rhodamine 6G (R6G) as a chemical Raman reporter and a Raman enhancement of 1.43 × 10^7^, thereby surpassing that achieved by the previous study by a factor of 100.^9^ Furthermore, we developed an *in situ* HKUST-1 nanoplate with HKUST-1/Cu(OH)_2_ for trimesic acid (BTC) SERS detection to validate the applicability of these heterojunctions to other types of MOFs. We elucidated the mechanism of these heterojunction structures, highlighting the role of excitons in facilitating efficient CT within the MOFs and hydroxide heterojunctions in addition to that of conventional PICT between MOFs and analytes.^37,38^ In other words, the heterojunction provides more electrons for the MOFs–analytes CT and further enhances the SERS intensity. The introduction of the heterojunction does not demolish the tailorability of MOFs themselves. We also showcased the practical application of the MOF nanoplates by successfully detecting the adulteration of omeprazole. The nanoplates also have high anti-interference ability and stability. Our approach offers a wide range of customization options for MOFs-based SERS detection for future research and applications in other areas.

## Results

### Fabrication of *In Situ* ZIF-67 Nanoplates

**Figure 1:**
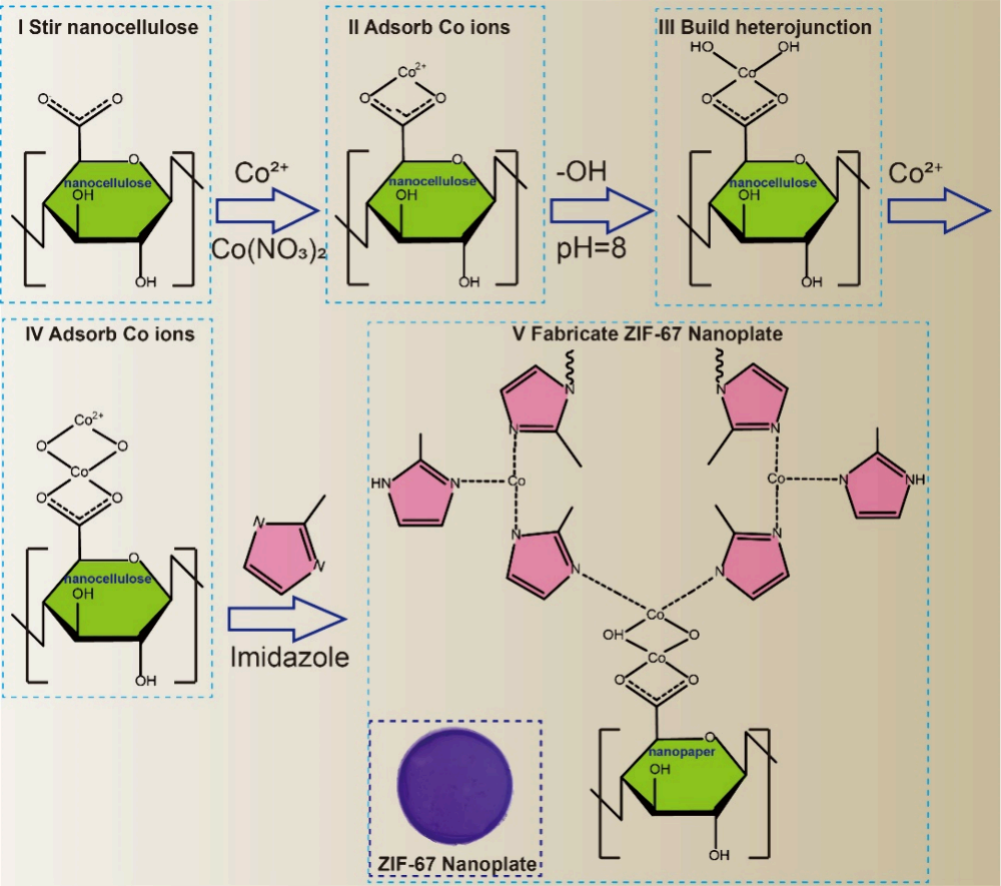
(I–V) Chemical schematic of the *in situ* ZIF-67 nanoplate fabrication process.

We employed a coordination replication technique to establish a simple and scalable fabrication process for the *in situ* ZIF-67 nanoplate. The process involved five main steps:

(I) The (2,2,6,6-tetramethylpiperidin-1-yl)oxyl (TEMPO)-oxidized nanofibrillated cellulose (NFC) slurry (0.1 wt % in distilled water) was stirred vigorously. (II) Co(NO_3_)_2_ was added and thoroughly mixed with the suspension, thereby facilitating the efficient adsorption of Co ions onto the nanopaper surface through electrostatic interactions with carboxyl groups. (III) The pH of the suspension was adjusted to ∼8.0 to build a Co(OH)_2_ heterojunction at the bottom layer. (IV) Excess Co ions were connected to the hydroxyl groups by complete stirring and incubation for 2 and 1 h, respectively. (V) The above suspension was vacuum filtered and washed with 50 mL of methanol to achieve nanopaper gel (4 cm in diameter) and further form the *in situ* ZIF-67 nanoplate. The gel was soaked in a 1,2-dimethylimidazole/methanol solution. After treatment with the imidazole solution, the shape of the upper layer was replicated to develop and anchor the ZIF-67 nanocrystals. Figure 1 and Figure S1 show the chemical and experimental schematic diagrams of the fabrication process, respectively. The obtained *in situ* ZIF-67 nanoplates were prepared for subsequent SERS detection.

### Characterization of the *In Situ* ZIF-67 Nanoplate

Several characterizations were performed to confirm the successful generation of the *in situ* ZIF-67/Co(OH)_2_ heterojunction structure on the nanopaper. X-ray photoelectron spectroscopy (XPS) and electronic paramagnetic resonance (EPR) spectroscopy were used to investigate the electronic structure and chemical bonding differences.

**Figure 2:**
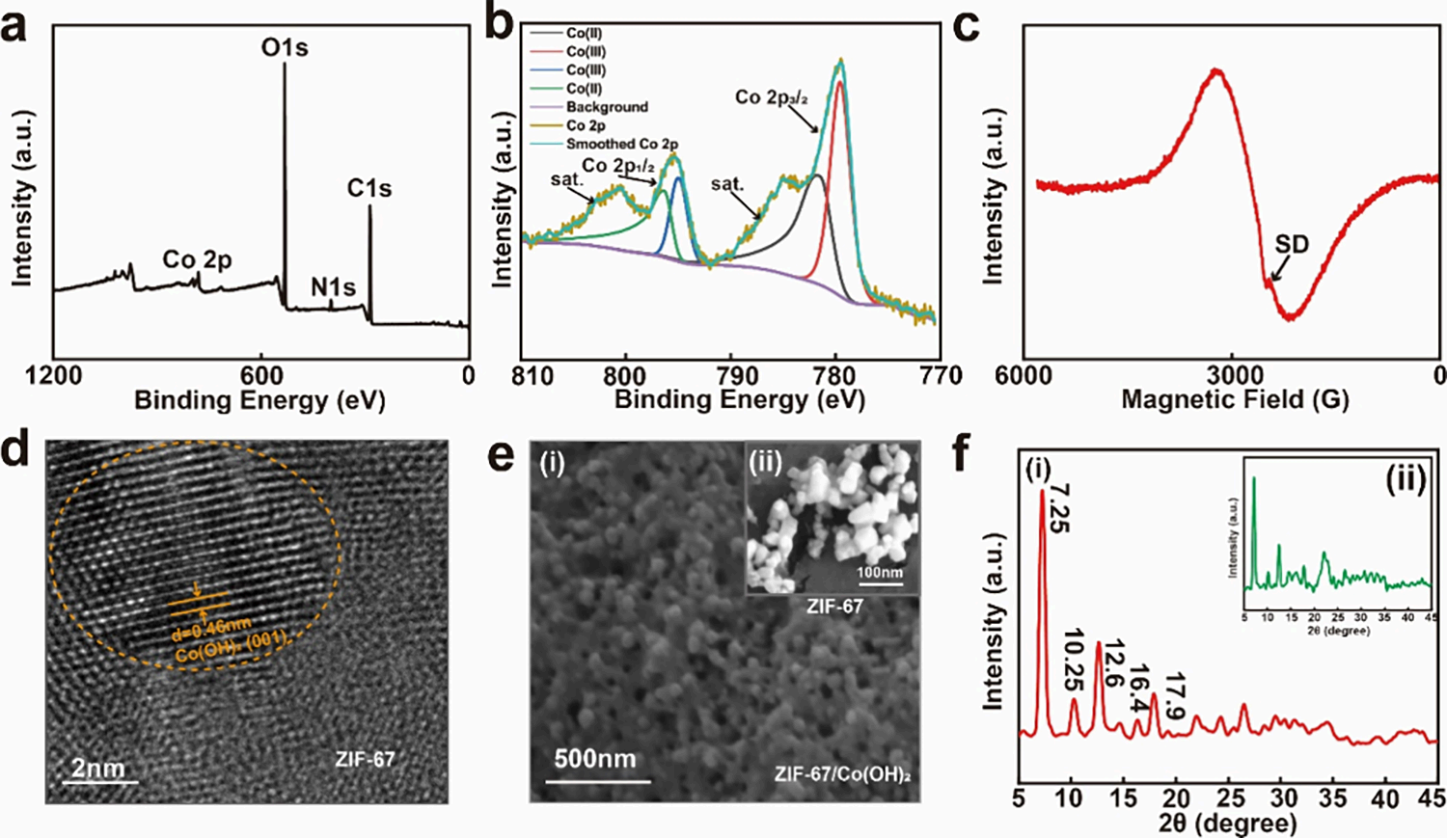
Characterization of the *in situ* ZIF-67 nanoplate. (a) XPS spectrum of the ZIF-67/Co(OH)_2_ structure. (b) XPS spectra of the central metal Co. (c) EPR spectrum of Co. (d) TEM image of the ZIF-67/Co(OH)_2_ structure. (e) SEM images of the (i) ZIF-67/Co(OH)_2_ structure and (ii) pure ZIF-67 on nanopaper. (f) XRD spectra of the (i) ZIF-67/Co(OH)_2_ lattice and (ii) pure ZIF-67 lattice on nanopaper.

Figure 2a shows the total spectrum of the element content and valence states of the *in situ* ZIF-67 nanoplate, whereas Figure 2b presents the XPS spectra of Co. The emergence of the additional peaks of Co and N, as well as those of C and O originally present in the nanopaper, indicates the formation of ZIF-67 nanocrystals on the nanopaper fibrous surfaces.^39^ The Co 2p3/2 peak, which is a characteristic peak of ZIF-67, was produced by the Co(II) species, with shakeup satellite peaks (hereafter, Sat.) at 781.8 and 796.5 eV. In contrast, the typical Sat. of Co(II) at 795.4 nm remained,^40^ with a small amount of Co(III) appearing with lower binding energies at 779.6 and 795.1 eV. The peak of Co(II) shifted from 785.2 eV (pure ZIF-67) to 781.8 eV, which illustrates the binding energy changes of our structure and provides further evidence of the successful formation of Co(OH)_2_ heterojunction. EPR is employed to detect valence changes in the metal, as indicated in Figure 2c. The EPR spectra comprise two broad components with average *g* factors of 2776.0 and 3540.0, indicating different valence states of the Co ions.

Transmission electron microscopy (TEM) images can effectively illustrate the heterojunction structures. A TEM image of the synthesized *in situ* ZIF-67/Co(OH)_2_ structure is shown in Figure 2d. The lattice spacing for Co(OH)_2_ is 0.46 nm, which corresponds well with the (001) plane.^41,42^ ZIF-67 exhibited a disordered lattice structure, and Co(OH)_2_ was successfully formed; the clear lattice fringes indicate good crystallinity of the catalysts after calcination.

The corresponding X-ray diffraction (XRD) spectra were recorded to confirm the successful growth of ZIF-67 on the Co(OH)_2_ heterojunction. Figure 2f(i) shows the XRD patterns of *in situ* ZIF-67/Co(OH)_2_ with six different characteristic diffraction peaks at 7.3, 10.3, 12.6, 16.4, and 17.9°,^43^ which indicates the successful preparation of ZIF-67 with the crystal structure of the upper layer.^44^Figure 2f(ii) illustrates the XRD patterns of the pure ZIF-67 crystal for comparison. The diffraction peaks of the new crystalline materials showed deviations from those of ZIF-67 but remained the same, which indicates that the framework structures of the samples were mainly conserved. This comparison also demonstrates the presence of Co(OH)_2_. These aforementioned characterizations indicate the successful generation of the ZIF-67/Co(OH)_2_ heterojunction structure and demonstrate the suitability of the developed substrate for SERS detection.

Figure 2e(i),(ii) shows the scanning electron microscopy (SEM) images of the *in situ* ZIF-67 particles without and with the Co(OH)_2_ heterojunction, exhibiting average particle sizes of ∼120 ± 6.7 and 50 ± 4.6 nm, respectively. These images confirm the successful growth of the ZIF-67 framework. Pure *in situ* ZIF-67 particles exhibited a low level of effectiveness, which was indicated by the small number of agglomerated particles. In contrast, the distribution of *in situ* ZIF-67 particles with the Co(OH)_2_ heterojunction appeared to be more uniform and continuous. Further, Figure 2e(i),(ii) reveals the structural transformation of ZIF-67 with a Co(OH)_2_ heterojunction from a mesoporous to a microporous structure. A narrower pore size distribution can minimize adsorbent blockage and reduce the effect on the adsorption of trace analytes.

The reaction process of color change can be easily monitored by the naked eye, as shown in Figure S2a,b, wherein the *in situ* ZIF-67 nanoplate exhibited a more intense color compared to pure ZIF-67 on nanopaper. The structure of the *in situ* ZIF-67 nanoplate is shown in Figure 1, where the carboxyl groups of the nanopaper adsorb Co ions, forming a layer of Co(OH)_2_, and hydroxyl bridges with excess Co ions generate ZIF-67 in the upper layer. These characterizations collectively confirm the successful development of the novel ZIF-67/Co(OH)_2_ heterojunction structure on the nanopaper.

### Optimization and SERS Detection Results of the *In Situ* ZIF-67 Nanoplate

**Figure 3:**
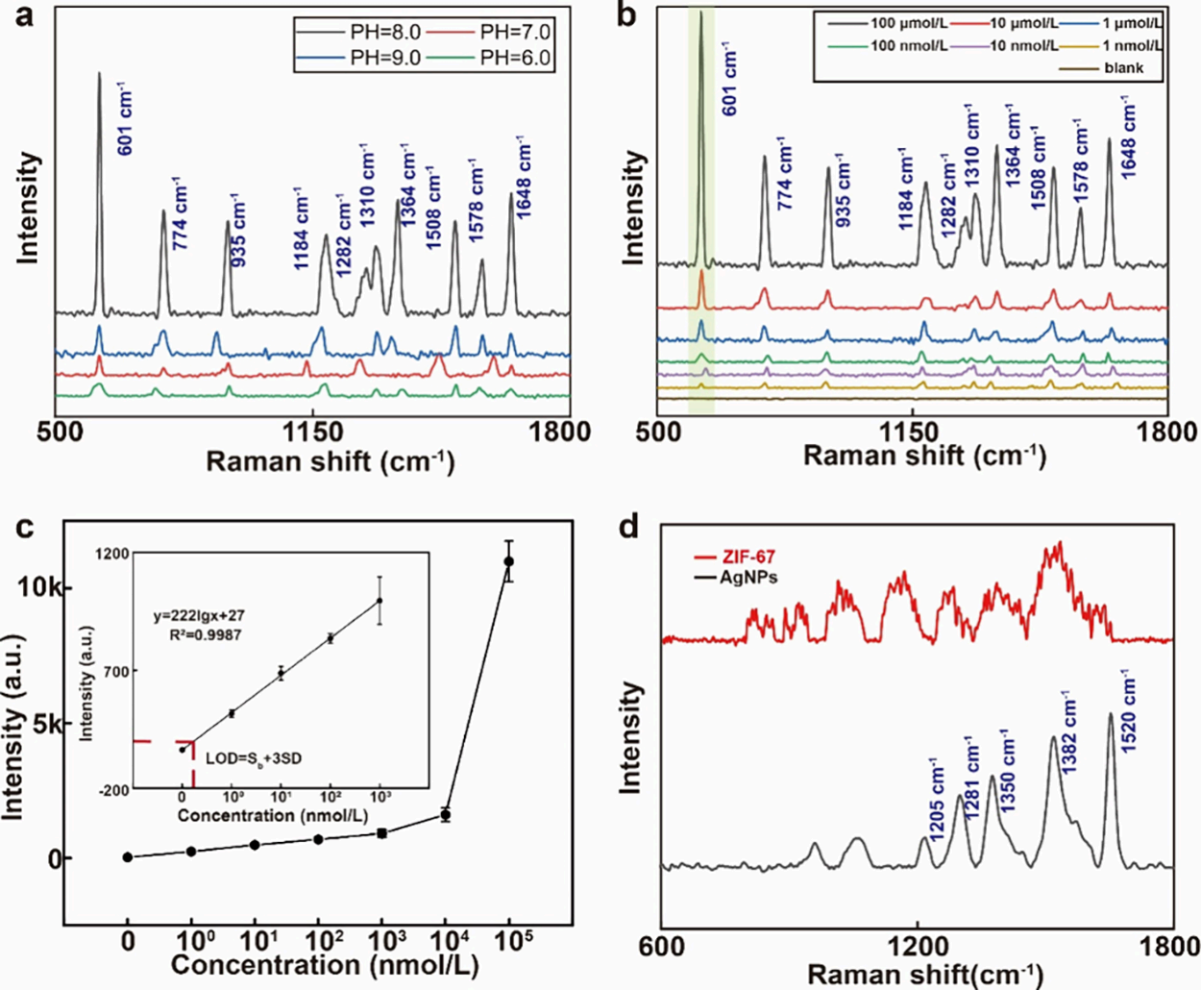
SERS detection on an *in situ* ZIF-67 nanoplate. (a) Optimization of the *in situ* ZIF-67 nanoplate. (b) Raman spectra of R6G at concentrations of 1 nmol/L to 100 μmol/L. (c) Calibration of R6G at 611 cm^–1^ (*n* = 5). (d) Comparison of the RhB Raman spectra on the *in situ* ZIF-67 nanoplate and AgNP substrate.

SERS enhancement is significantly affected by the effective generation of heterojunctions.^25,38^ A binding energy approaching the center of ZIF-67 and Co(OH)_2_ can provide a higher SERS signal because it indicates a combination of both ZIF-67 and Co(OH)_2_ instead of only one material.^45^ Thus, the binding energy of the ZIF-67/Co(OH)_2_ heterojunction structure can affect the SERS intensity. We verified this using a common environmental pollutant R6G (1 × 10^–4^ mol/L R6G solution) as a sensing example. Here, 4.0 mg/mL NaOH solution was added in varying volumes of 0.0, 2.0, 4.0, and 6.0 mL, corresponding to pH values of 6.0, 7.0, 8.0, and 9.0, respectively. Figure 3a shows that the characteristic peak of R6G at 611 cm^–1^ had the highest intensity at pH = 8.0, whereas the characteristic peak of the tested substance could not be effectively detected at pH = 10.0. Excessive hydroxide leads to the formation of cobalt hydroxide precipitates, thereby preventing the adsorption of additional Co ions necessary for generating ZIF-67 and hindering SERS enhancement. Insufficient hydroxide results in the absence of ZIF-67/Co(OH)_2_ heterojunction materials, and SERS enhancement relies solely on the charge mechanism of ZIF-67. The value at pH = 8.0 is close to the median, thereby providing a higher SERS intensity. Therefore, 4.0 mL of a 4.0 mg/mL NaOH solution was selected for further experiments.

The detection capability of the optimized *in situ* ZIF-67 nanoplate was evaluated via Raman spectroscopy measurements on R6G samples with concentrations ranging from 1 × 10^–4^ to 1 × 10^–9^ mol/L in ethanol, wherein pure ethanol served as a blank control (Figure 3b). The measured spectra exhibited strong R6G bands, including C–C ring bending (610 cm^–1^), C–H out-of-plane bending (774 cm^–1^), C–H bending (935 cm^–1^), C–O–C stretching (1184 and 1282 cm^–1^), and aromatic C–C stretching (1310, 1364, 1508, 1578, and 1648 cm^–1^).^46^ We selected the 610 cm^–1^ peak intensity as the reading because it exhibits the average of the five readings shown in Figure 3c. The LOD was calculated to be 0.98 nmol/L, which is defined as the R6G concentration that corresponds to the intensity of the blank control plus three times the standard deviation of the Raman intensity of the blank control. The linear regression equation was *y* = 222lg*x* + 27, and the correlation coefficient (*R*^2^) was 0.9987. Therefore, the detection limit was as low as 1 × 10^–10^ mol/L, which is evidently lower than that of the untreated ZIF-67 sample. The coefficient of variation of R6G on this nanoplate is shown in Table S2.

To evaluate the SERS activity on the ZIF-67 plate, the Raman enhancement factor (EF) was calculated from the R6G detection results using , where *I*_SERS_ and *C*_SERS_ represent the intensities of the Raman spectra at 610 cm^–1^ with the SERS effect and the concentrations on the *in situ* ZIF-67 nanoplate, respectively. *I*_bare_ and *C*_bare_ represent the same on bare nanopaper, respectively. The calculated EF for the *in situ* ZIF-67 nanoplate was 1.43 × 10^7^, which is comparable to the performance of modern inorganic semiconductors and noble metals, thereby confirming its high SERS sensitivity toward R6G. The LOD achieved for the *in situ* ZIF-67 nanoplate was 100 times better than that reported previously (10^–7^ mol/L).^9^ Also, compared to the EF of the pure ZIF-67 substrate, our method achieves a 10 times increase. We further evaluated the stability testing SERS results after 1 week, 2 weeks, and 1 month of storage at room temperature, finding consistent results compared to the freshly prepared samples (Figure S3). With the growth of storage time, the intensity of R6G detection decreased slowly (∼4.50%), and the noise of the characteristic peaks increased (1282 to 1648 cm^–1^ peaks). Also, the *in situ* ZIF-67 nanoplate was still detectable after storing for one month.

Two different Raman reporters were illustrated using an *in situ* ZIF-67 nanoplate as an example to demonstrate the tailorability of ZIF-67 nanoplates. When R6G was employed as the target molecule, the highest occupied molecular orbital (HOMO) and lowest unoccupied molecular orbital (LUMO) levels were −5.7 and −3.4 eV, respectively.^47^ The HOMO of ZIF-67 is −5.8 eV, and HOMO-to-LUMO charge-transfer transitions between ZIF-67 and R6G exist in ZIF-67 under 532 nm laser excitation. Figure 3d shows the Raman spectra of RhB (10^–5^ mol/L) when excited with a 532 nm laser. In contrast, with a 532 nm laser stimulation, the SERS effect on the *in situ* ZIF-67 nanoplate for RhB was undetectable because of the mismatch between the HOMO (−3.4 eV) and LUMO (−2.8 eV) levels.^48,49^ The anti-interference ability of the *in situ* ZIF-67 nanoplate is shown in Figure S4, while the solution with R6G and RhB at the same concentration (10^–5^ to 10^–9^ mol/L) was detected. Due to the energy level matching principle, only R6G*'*s characteristic peaks were analyzed, which illustrates the anti-interference ability of our method (Figure S4). This observation indicates that the developed MOFs/heterojunction (M_*x*_(OH)_*y*_-assisted) retained the tailorability of the MOFs, with the charge mechanism playing a central role in the SERS enhancement. This *in situ* ZIF-67 nanoplate suggests that the structural engineering of MOFs can provide exceptional selectivity for SERS, thereby enabling the analyte-oriented customization of SERS substrates. Further, this LOD specificity is less frequently observed for other SERS substrates due to the evenness for MOFs with substantial structural flexibility. This unique characteristic of MOFs suggests that they can achieve good selectivity in SERS, which is important for practical analytical applications.

### Raman Enhancement Mechanisms

**Figure 4:**
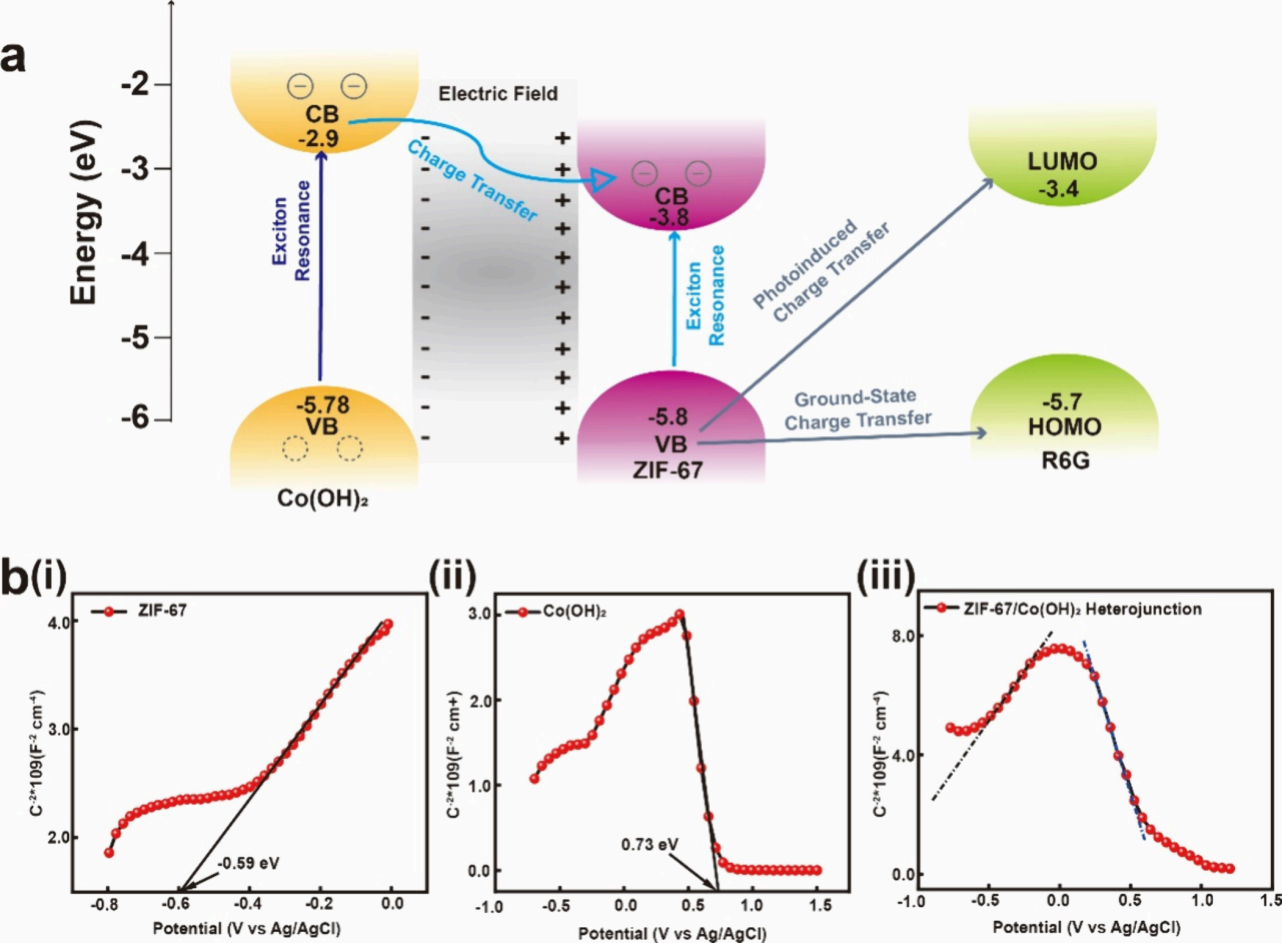
SERS mechanisms of *in situ* ZIF-67 nanoplate. (a) Energy level diagram of R6G molecule under the illumination of 532 nm. (b) Mott–Schottky analysis of (i) ZIF-67, (ii) Co(OH)_2_, and (iii) ZIF-67/Co(OH)_2_ heterojunction.

Further investigations were conducted to provide more detailed explanations of the underlying mechanisms. EM and CM are commonly acknowledged as the two main mechanisms of SERS.^50^ Cong et al. indicated that the CT resonance between the band edges of the substrate and the affinity levels of the adsorbed analyte play a key role in SERS enhancement.^9^ Semiconductor SERS substrates have been proven to show Raman enhancement by CM, and EM only realizes on semiconductor substrates in longer wavelength regions (infrared wavelength).^51^ Since the excitation wavelength for ZIF-67 is 532 nm, which is much smaller than the infrared wavelength, only CM works in our system. The exceptional optical and structural characteristics of semiconductor–heterojunctions have led to their extensive application in solar cells, photocatalysis, and gas sensing.^52,53^ The reason for this is that the heterojunctions that develop at the interface have the ability to separate electron–hole pairs, hence reducing electron–hole recombination and improving the usage of photoinduced electrons.^54^ The charge-transfer theory leads us to suppose that these kinds of semiconductor–heterojunctions may also help PICT in semiconductor–molecule systems, which could enhance the semiconductor’s SERS activity. Under appropriate laser excitation, two possible HOMO-to-LUMO CT transitions can occur in the MOFs, and MOFs that rely only on the chemical mechanism of SERS enhancement can achieve an LOD of 10^–7^ mol/L.^9,20^ Therefore, introducing heterojunctions significantly improves the performance as it enables incorporation of additional charge transfer. A schematic of the energy levels of the *in situ* ZIF-67 nanoplate-molecule system under 532 nm laser illumination is shown in Figure 4a to further elucidate the role of the heterojunction in the PICT process. The HOMO and LUMO levels of the R6G probe are −5.7 and −3.4 eV, respectively. For the Co(OH)_2_-R6G system, the excitation energy is insufficient to perform CT from the HOMO of R6G (−5.7 eV) to the conduction band (CB) of Co(OH)_2_ (−2.9 eV) or from the valence band (VB) of Co(OH)_2_ (−5.78 eV) to the LUMO of R6G (−3.4 eV).^55^ A previously reported semiconductor-to-molecule CT mechanism was followed for the ZIF-67-R6G system. In short, the electrons in the VB of ZIF-67 (−5.80 eV) were injected into the LUMO of the R6G molecules (−3.4 eV). The standard formula for the polarization tensor is given by *A* + *B* + *C*, where *A* represents the molecular resonance and *B* and *C* represent the two charge-transfer resonances.^56,57^ The SERS caused by the CT effect has the potential to theoretically borrow intensity from the Albrecht *B* and *C* terms, which are vibronic coupling terms. The enhanced contributions from semiconductor-to-molecule and molecule-to-semiconductor charge-transfer transitions are represented by the *B* and *C* terms, respectively. CT transitions from the semiconductor to the molecule take place in this work.^58^ However, *B* and *C* were not realized in the Co(OH)_2_-R6G system, so Co(OH)_2_ only provides an additional charge for the ZIF-67-R6G system.

Under a 532 nm laser excitation, the heterojunction exhibits exciton resonance induced by the electron transition between the VB and CB states in Co(OH)_2_.^59−61^ The photoexcited electrons in Co(OH)_2_ within the heterojunction are transported to the CB of ZIF-67, thereby supplying additional electrons to the LUMO of R6G. Consequently, the PICT resonance between the ZIF-67/Co(OH)_2_–R6G ternary system is enhanced because of the novel exciton resonance in the monolayer Co(OH)_2_ compared to that in the ZIF-67-R6G system alone. Therefore, the PICT efficiency of the ZIF-67/Co(OH)_2_–R6G system is effectively increased by CT facilitated by Co(OH)_2_. These CT resonances significantly increase the polarization tensor of the substrate, enhance the Raman scattering cross-section, and contribute to the overall enhancement of the Raman signals in the heterojunction under 532 nm illuminated laser.^56,62^ Strength in heterojunction is a crucial factor for achieving this enhanced PICT effect.^63^ These findings underscore the importance of strong interfacial coupling in heterojunctions to achieve a superior SERS performance.

Mott–Schottky plots were employed to investigate the electron transport behavior of the synthesized polyhedron catalysts in a 0.5 mol/L Na_2_SO_4_ electrolyte at a frequency of 1000 Hz. Figure 4b shows the Mott–Schottky spectra of ZIF-67, Co(OH)_2_, and ZIF-67/Co(OH)_2_. Figure 4b(i),(ii) shows that ZIF-67 and Co(OH)_2_ have positive and negative slopes of the E–C^2–^ plots, respectively, indicating their n-type and p-type semiconductor characteristics.^64^ When p- and n-type semiconductors are joined to create a heterojunction, the offset of the Fermi levels transports electrons from the n-type semiconductor to the p-type semiconductor.^65−67^ Consequently, the n-type semiconductor side becomes positively charged, whereas the p-type semiconductor side becomes negatively charged, thereby resulting in the development of an electric field.^68^ The spectrum of the ZIF-67/Co(OH)_2_ heterojunction structure shows the presence of both n- and p-type semiconductors, further confirming the SERS enhancement mechanism.

### Characterization of the *In Situ* HKUST-1 Nanoplate

To expand the above findings to other types of MOFs, HKUST-1 was chosen to develop the SERS substrate since HKUST-1 can grow on nanopaper by a facile absorption and reduction reaction. The HKUST-1/Cu(OH)_2_ structure was presented to verify the feasibility of generating a specific MOF/hydroxide heterojunction structure in other types of MOFs. *In situ* HKUST-1 nanoplate fabrication was similar to that of ZIF-67, although the pH was adjusted to 8.0 for further experiments. Several characterizations were performed as follows.

**Figure 5:**
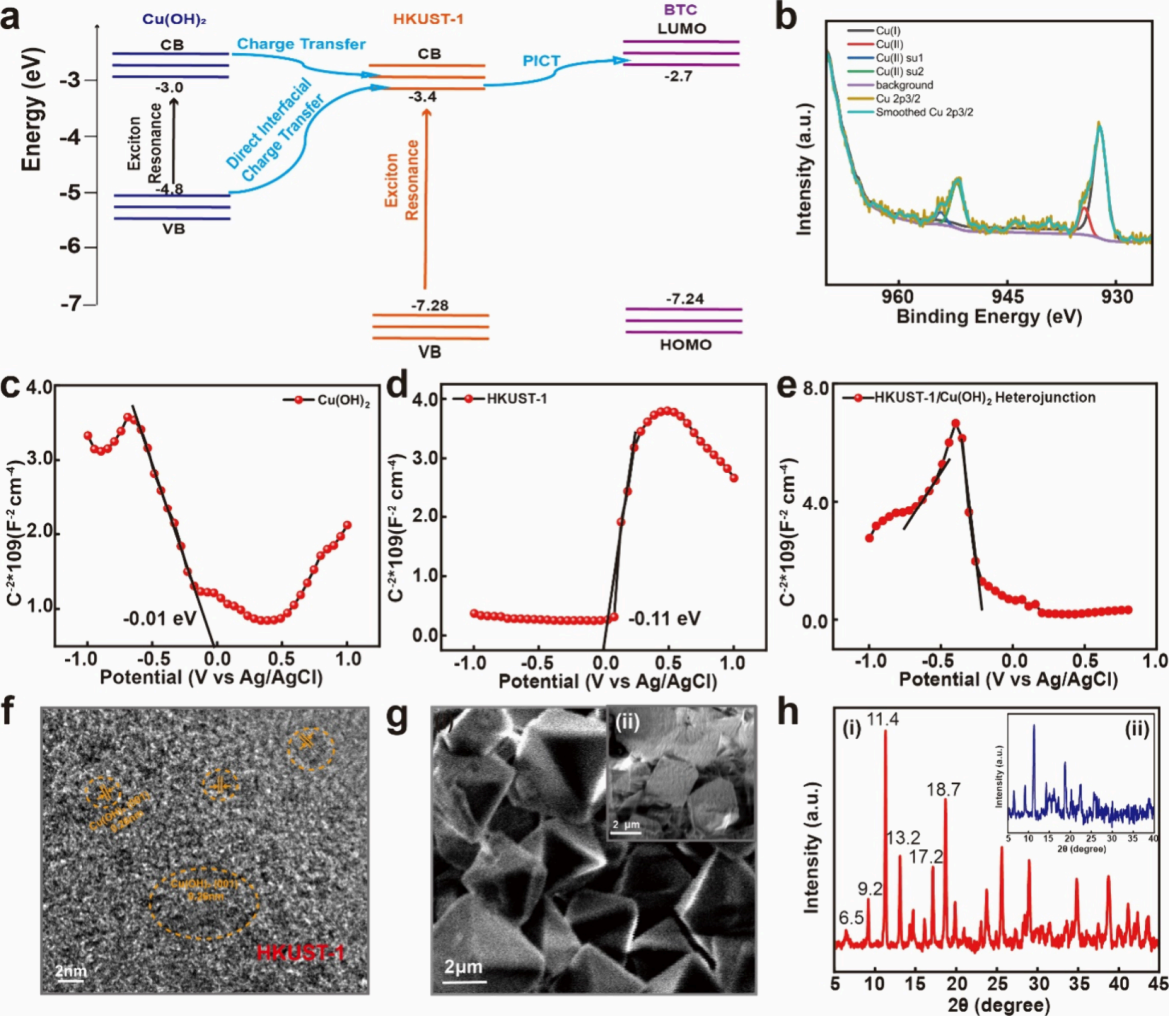
Characterization of the *in situ* HKUST-1 nanoplate. (a) Energy level diagram of the BTC molecule on *in situ* HKUST-1 nanoplate under a 532 nm laser illumination. (b) XPS spectra of central metal Cu. (c–e) Mott–Schottky analysis of Cu(OH)_2_, HKUST-1, and HKUST-1/Cu(OH)_2_, respectively. (f) TEM image of the HKUST-1/Cu(OH)_2_ heterojunction. (g) SEM images of the (i) HKUST-1/Cu(OH)_2_ heterojunction structure and (ii) HKUST-1 on nanopaper. (h) XRD spectra of the (i) HKUST-1/Cu(OH)_2_ heterojunction and (ii) HKUST-1 lattice.

The energy level diagram of the ethanol molecule on an *in situ* HKUST-1 nanoplate under illumination of 532 nm is shown in Figure 5a. The dimeric form of BTC is a common structure that of its stable monomer structure by high-frequency O–H stretching and low-frequency O–O stretching mode. The HOMO and LUMO levels of dimeric BTC are −7.24 and −2.7 eV, respectively. For the Cu(OH)_2_–BTC system, the excitation energy is insufficient to carry out CT from the HOMO of BTC (−7.24 eV) to the CB of Cu(OH)_2_ (−3.0 eV) or the VB of Cu(OH)_2_ (−4.8 eV).^69,70^ The HOMO and LUMO of BTC contain the energy level of Cu(OH)_2_, so there is no SERS effect. The HKUST-1-BTC system followed a previously reported semiconductor-to-molecule CT mechanism. That is, charges in the VB of HKUST-1 (−7.28 eV) are injected into the LUMO of BTC.^71,72^Figure 5c–e shows the Mott–Schottky spectra of HKUST-1, Cu(OH)_2_, and HKUST-1/Cu(OH)_2_, respectively. As displayed in Figures 5c–d, HKUST-1 and Cu(OH)_2_ show positive and negative slopes of the E–C^2–^ plots, respectively, which manifest the characteristics of n- and p-type semiconductors. The spectra of the HKUST-1/Cu(OH)_2_ heterojunction structure showed both n- and p-type semiconductors, further confirming the SERS enhancement mechanism described earlier.

Figure 5b shows the XPS profiles, which indicate the valence states of Cu. The peaks at 932.2 and 934.5 eV are attributed to Cu(I) and Cu(II), respectively. The Cu(II) oxidation state is linked to two peaks at 945.7 and 938.5 eV.^73^ The Cu(II) peak shifted from 931.2 eV (pure HKUST-1) to 932.2 eV, illustrating the electron transfer from Cu(OH)_2_ to HKUST-1 and confirming the successful generation of the heterojunction. These occur when the Cu electronic structure is unsaturated (d^9^), thereby permitting p–d hybridization. Such features were not present in the Cu(I) oxidation state because the electronic configuration was d^10^ and only one peak was visible in the 2p3/2 region. Peak fitting was used to determine the relative abundances of Cu(I) and Cu(II) using the Cu 2p3/2 signal and its shakeup characteristics. The Cu 2p3/2 spectrum in Figure 5b demonstrates the coexistence of Cu(I) and Cu(II) in the framework.^74^

The XRD patterns of the samples are shown in Figure 5h(i). The diffraction peaks at 2θ = 6.5, 9.2, 11.4, 13.2, 17.2, and 18.7° show the successful synthesis of the high-purity HKUST-1/Cu(OH)_2_ framework.^75^ Compared with the XRD peaks of HKUST-1 in Figure 5h(ii), the diffraction peaks of HKUST-1/Cu(OH)_2_ differ from those of HKUST-1 but remain identical to them, indicating that the framework structures of the samples were mostly preserved.

Figure 5f depicts a TEM image; a lattice fringe of 0.26 nm is assigned to the (001) facet of Cu(OH)_2_.^76^ Furthermore, growing SERS platforms contribute to highly sensitive detection. Figure S6a shows a photograph of the *in situ* HKUST-1 nanoplate prepared for testing. Further, we characterize the framework using SEM images (Figure 5g(i)) and compare it with that of pure HKUST-1 on nanopaper (Figure 5g(ii)). Dense, uniform, and well-organized arrays of HKUST-1 were formed with an average 2 μm diameter, thereby illustrating the successfully growing framework. All characterizations illustrate that the developed *in situ* HKUST-1 nanoplate can be used for highly sensitive SERS detection.

### SERS Detection Results of the *In Situ* HKUST-1 Nanoplate

**Figure 6:**
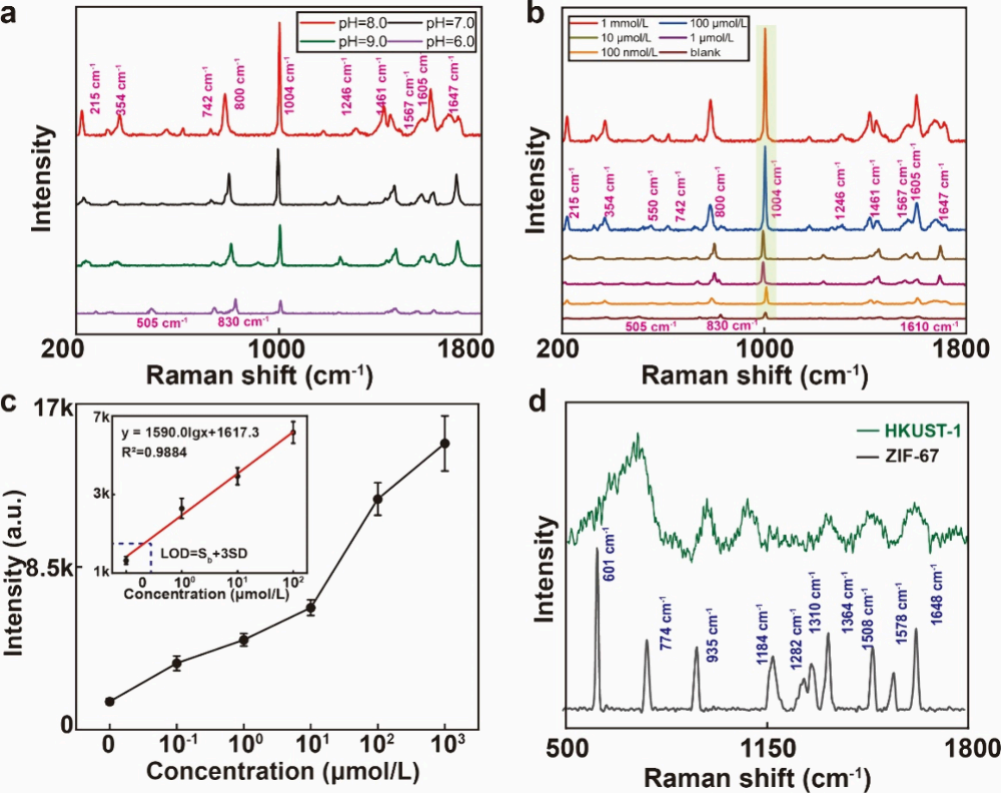
SERS detection on the HKUST-1 nanoplate. (a) Optimization of the *in situ* HKUST-1 nanoplate. (b) Raman spectra of BTC at concentrations of 10^–3^ to 10^–8^ mol/L. (c) Calibration of ethanol at 1004 cm^–1^ (*n* = 5). (d) Raman spectra of R6G on ZIF-67 and an *in situ* HKUST-1 nanoplate.

BTC was used as the Raman reporter to validate the effectiveness of the developed *in situ* HKUST-1 nanoplate for SERS detection. Figure 6a shows that the characteristic peaks of BTC under the same concentration had the highest intensity at pH = 8.0. Similar to the ZIF-67 nanoplate, excessive hydroxide leads to the formation of cobalt hydroxide precipitates, thereby preventing the adsorption of additional Cu ions necessary for generating HKUST-1. On the contrary, a small amount of alkali cannot form an effective heterojunction. Also, under pH 6.0, the SERS intensity of BTC is quite low and is closer to the peak of only HKUST-1. Therefore, pH = 8.0 was selected for further experiments.

Figure 6b shows the Raman spectra of BTC samples in infant formulas at different concentrations (from 10^–3^ to 10^–7^ mol/L). These spectra exhibit characteristic Raman bands of ethanol at 215 cm^–1^ (−COOH), 354 cm^–1^ (C–O–H), 550 cm^–1^ (H–H), 742 cm^–1^ (O–C–O), 800 cm^–1^ (CH), 1004 cm^–1^ (benzene ring), 1246 cm^–1^ (CH_3_ rocking), 1461 and 1567 cm^–1^ (CH_3_ bending), 1605 cm^–1^ (C–O), and 1647 cm^–1^ (O–H).^77^ The band at 1647 cm^–1^ is assigned to the C=O stretching accompanied with the O–H in-plane bending under a strong dimer structure.^78^ Compared to BTC, the peaks of HKUST-1 shift from 800 to 830 cm^–1^ and 1605 to 1610 cm^–1^ ascribed to out-of-plane ring (C–H) bending vibrations and ν(C=C) modes of the benzene ring.^79^ Also, 270 and 505 cm^–1^ are Cu–O peaks from HKUST-1.^80^ Among these peaks, the peak at 1004 cm^–1^ was found to be the most prominent and selected as the reading for the concentration analysis. The concentration-dependent SERS intensity at this peak can be described by *y* = 1590.0lg*x* + 1617.3, where *x* represents the BTC concentration in water. The LOD for BTC was estimated as 15.4 nmol/L (Figure 6c) with 0.9884 *R*^2^. The coefficient of variation of BTC on this nanoplate is shown in Table S4. These results collectively indicate that the defects of the MOF contribute to good stability and enable the observed SERS behavior, thereby providing a significant advantage over other semiconductors. For comparison, the *in situ* HKUST-1 nanoplates are added with 5 μL 10^–4^ M R6G solution. Figure 6d illustrates the Raman spectrum of R6G at 532 nm. No detectable SERS effect is observed on the *in situ* HKUST-1 nanoplate for 10^–4^ M R6G, which can be attributed to the mismatch between the energy levels of HKUST-1 and R6G, thereby hindering CT. Similar to ZIF-67 nanoplates, HKUST-1 nanoplates also exhibit high stability. The SERS detection results in a 1 week interval at 10^–5^ mol/L are provided in Figure S6, while the peaks at 812 cm^–1^ decrease tremendously around 50% and 830 cm^–1^ appeared after 1 month of storing. Also, the intensity of Cu–O (270 and 505 cm^–1^) peaks increases, which illustrates that more Cu was oxidized. Achieved by combining resonant transitions, the observed selectivity in the SERS detection of various analytes on various MOFs is attributed to band-level alignment based on the wide range of band structures found in MOFs.

### Detecting Adulteration in Medicine

Medical detection was considered as an example to demonstrate the practical applications of *in situ* MOFs/M_*x*_(OH)_*y*_ nanoplates in daily life. Gastroesophageal reflux disease and erosive esophagitis, caused by the reflux of acidic gastric content, have become increasingly prevalent because of rising social pressures and irregular lifestyles.^81−83^ Omeprazole is a proton-pump inhibitor acting on gastric parietal cells to limit gastric acid production and is commonly used to treat gastric diseases.^84^ According to a report by the World Health Organization, approximately 50% of the medicines traded on Internet websites are counterfeit.^85^ Many research shows that a semiconductor SERS substrate can effectively be used for drug detection.^86,87^ We demonstrate the flexible SERS detection capabilities on our nanoplates for omeprazole and compare the characteristic peaks of genuine and counterfeit drugs (starch) to facilitate market supervision and reduce losses.

**Figure 7:**
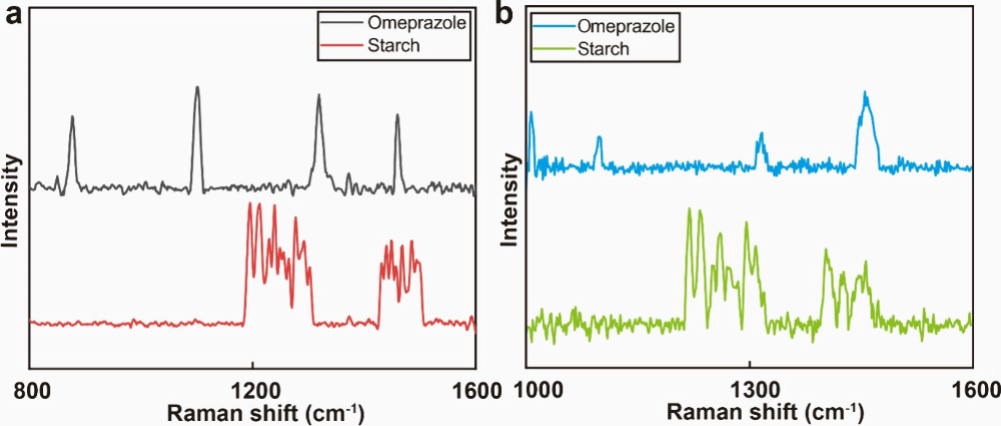
Raman spectra of pure and fake omeprazole (a) on the *in situ* ZIF-67 nanoplate and (b) the *in situ* HKUST-1 nanoplate.

The charge level of omeprazole (HUMO (−5.5 eV) and LUMO (−0.7 eV)) is within the detection limits of the ZIF-67 nanoplate, whereas the HUMO and LUMO of the counterfeit drug cannot satisfy the charge level.^88^Figure 7 shows the Raman spectra of omeprazole and starch samples at 3.0 mg/mL in ethanol, which is the minimum concentration of drugs sold on the market: the measured spectra contain strong omeprazole bands at 880 cm^–1^ (the stretching of the ether groups presents in the two aromatic rings of the omeprazole molecule), 1100 cm^–1^ (the stretching of the C–C), 1322 cm^–1^ (the symmetric stretching of the chain vibrations of the two aromatic rings), 1380 cm–1 (weak symmetric bending of methyl groups), and 1461 cm^–1^ (the asymmetric bending).^89^ Compared to dye molecules, omeprazole exhibits poorer adsorption on semiconductors and needs to be dropped multiple times until fully adsorbed. The spectra for the starch samples did not display the same characteristic peaks, providing ample evidence that our nanoplates can effectively distinguish genuine medicines from counterfeit ones. The charge level of omeprazole is also within the detection limits of the HKUST-1 nanoplate. Two types of *in situ* MOFs nanoplates that we designed can all be unitized for detecting adulteration in medicine. Due to the influence of adsorption, the intensity of omeprazole on HKUST-1 is relatively low. Thus, the unique structure of the MOFs/heterojunction structure helps ensure the quality of medicines and addresses the issue of counterfeit drugs. In the future, we plan to expand the assay applications to include the multiplexed detection of other analytes, such as proteins and other medications.^90,91^

## Conclusions

In this study, we developed a special MOF/heterojunction (M_*x*_(OH)_*y*_-assisted, M = Co, Cu, or other metals) structure to enhance the SERS performance. We demonstrated that the resonance between the incident light and heterojunction plays a crucial role in intense localized surface plasmon resonance. The heterojunctions not only generated near-field Raman enhancement but also facilitated charge transfer. Consequently, the efficiency of PICT between the semiconducting substrate and the molecules was significantly enhanced through direct interfacial CT charge transfer processes by heterojunction. We also demonstrated two different types of *in situ* MOF nanoplates (with ZIF-67/Co(OH)_2_ and HKUST-1/Cu(OH)_2_) and achieved an LOD of 0.98 nmol/L for an R6G Raman reporter with a Raman EF of 1.43 × 10^7^, which is 100 times better than the sensitivity achieved using a pure ZIF-67 substrate for SERS detection. The nanoplates that we developed still maintain the tailorability of MOFs. Further, we successfully detected adulteration in medicines for omeprazole detection on these two nanoplates. Our findings also provide a facile and stable substrate compared with other MOF-based substrates, further exploring the possibility of using MOFs for SERS detection. Additionally, for the first time, it was found that heterojunction structures can effectively enhance the signal of MOFs-based SERS detection. Our research highlights the potential of these special MOFs/heterojunction substrates for SERS detection and underscores the importance of achieving high degrees of diversity and customization. In the future, artificial neural networks could be introduced to MOFs-based SERS for multiple detection. The findings will open up new possibilities for sensitive and selective detection in various applications, including the detection of medicine adulteration.

## Methods

### Reagents and Materials

TEMPO-oxidized NFC slurry (1.0 wt % solid, carboxylate level 2.0 mmol/g solid, average nanofiber diameter: 10 nm) was purchased from Tianjin University of Science and Technology (Tianjin, China). R6G (AR), RhB (>98%), methanol (>99.5%), BTC (>98%), Na_2_SO_4_ (>99%), and Cu(NO_3_)_2_·3H_2_O (>99%) were obtained from Macklin (Shanghai, China). Co(NO_3_)_2_·6H_2_O (>99%), NaOH (>97%), and 1,2-dimethylimidazole (>98%) were purchased from Aladdin (Shanghai, China). Ethanol (>99%) was ordered from Hushi (Shanghai, China).

### Preparation of the *In Situ* ZIF-67 Nanoplate

Here, 4.0 g of TEMPO-oxidized NFC slurry was dispersed in distilled water to a final content of 0.1 wt %, and the suspension was stirred sufficiently. Three grams of Co(NO_3_)_2_·6H_2_O was added to the above suspension until fully mixed. Four milliliters of NaOH solution (4 mg/mL) was gradually dropped into the mixed suspension. The mixture was stirred at 1000 rpm for 2 h and stewed for another 1 h for adequate adsorption. The prepared suspension was vacuum filtered on a glass filter holder with a PVDF filter membrane (VVLP04700, EMD Millipore Corporation, pore size: 0.1 μm). Then, 100 mL of methanol was used for washing three times. Next, the filtered nanopaper film was soaked in 50 mg/mL 1,2-dimethylimidazole/methanol solution for 12 h. The prepared film was fully washed in methanol for further application.

### Preparation of the *In Situ* HKUST-1 Nanoplate

Four grams of TEMPO-oxidized NFC slurry was dispersed in distilled water to a final content of 0.1 wt %, and the suspension was stirred sufficiently. Three grams of Cu(NO_3_)_2_·3H_2_O was added to the above suspension until fully mixed. NaOH solution was gradually dropped into the mixed suspension to adjust the pH to 8.0. The above solution was stirred at 1000 rpm for 2 h and stewed for another 1 h for adequate adsorption. Then, 50 mL of DI water and ethanol were used for washing by filtering, respectively. Next, the filtered nanopaper film was soaked in 25 mg/mL BTC solution (DI water/ethanol = 1:1) for 12 h. The prepared film was fully washed for further application.

### SERS Measurements

All Raman spectra were measured by the Renishaw Micro-Raman Spectroscopy System (U.K.) with a 532 nm laser and a 50× objective. R6G was dissolved in ethanol with concentrations ranging from 10^–4^ to 10^–9^ mol/L. Five microliters of the analyte solution was dropped on the *in situ* ZIF-67 nanoplate and further left to dry in the air. The Raman spectrum was acquired in the region of 500–1800 cm^–1^. Moreover, the 10^–4^ M ethanol was chosen to optimize the *in situ* ZIF-67 nanoplate. For the SERS detection of the *in situ* HKUST-1 nanoplate, the different concentrations of BTC (from 10^–3^ to 10^–7^ mol/L) were chosen as proof. The analyte solution was dropped on the *in situ* HKUST-1 nanoplate for full adsorption and was further dried in air. The Raman spectrum was acquired in the region 200–1800 cm^–1^. Raman spectra were taken from the average of five measurements. All spectral data were analyzed using the Origin Lab software (OriginLab, U.S.A).

### Photoelectrochemical Measurements

All photoelectron chemistry experiments were tested by an electrochemical workstation (Metrohm PGSTAT302N, Switzerland) in a homemade standard three-electrode cell. The reference electrode, counter electrode, and working electrode used were saturated calomel electrode, Pt electrode, and FTO (1 × 1 cm^2^) drop-coating homogeneous catalyst, respectively. The electrolyte is Na_2_SO_4_ aqueous solution (0.5 mol/L). The photocurrent current density–time curve has a test bias of 0 V. The linear scanning voltammetry test has a voltage sweep range of −1.0 to 1.0 V.

### Instrumentation

TEM images were obtained with transmission electron microscopy at a 50 kV accelerating voltage (FEI Talos F200X G2, U.S.A).

SEM (FEI Scios 2 HiVac, U.S.A.) was used to characterize the morphologies of ZIF-67 and HKUST-1 at a working voltage of 5 kV.

XPS (Thermo Scientific K-Alpha, U.S.A.) was used to calculate the element content and valence at a working voltage of 12 kV.

Electronic paramagnetic resonance was detected by EPR (Bruker EMXplus-6/1, Germany).

The crystal phase was characterized by diffraction X-rays (D8 ADVANCE, U.S.A).

All spectral data was analyzed using Origin Lab software (OriginLab, U.S.A).

## Supporting Information Available

Schematic diagram of the ZIF-67 nanoplate fabrication process; photographs of *in situ* ZIF-67 nanoplate with and without Co(OH)_2_ heterojunction; coefficients of variation of R6G SERS detection at different concentrations; SERS spectra of R6G on ZIF-67 stored for 1 week, 2 weeks, and 1 month; specific SERS detection of R6G and RhB solution on ZIF-67; photographs of the *in situ* HKUST-1 nanoplate with and without Cu(OH)_2_ heterojunction; coefficients of variation of R6G SERS detection at different concentrations; and SERS spectra of ethanol on HKUST-1 stored for 1 week, 2 weeks, and 1 month (PDF)
