# Supplementary material for: Metal–Organic Frameworks/Heterojunction Structures for Surface-Enhanced Raman Scattering with Enhanced Sensitivity and Tailorability
Source: ACS Appl Mater Interfaces. 2024 May 8;16(20):26374–85. doi: 10.1021/acsami.4c01588 (PMC11129117; doi:10.1021/acsami.4c01588)
Supplement: Supplementary file 1 — am4c01588_si_001.pdf [file am4c01588_si_001.pdf]

MOFs/Heterojunction Structures for Surface-enhanced Raman Scattering with Enhanced Sensitivity and Tailorability

Wenwen Yuan,[1, 2, 3,#] *Keran Jiao,*[1, 2,#] *Hang Yuan,*[1] *Hongzhao Sun,*[4] *Eng Gee Lim,*[1, 2] *Ivona Mitrovic,*[2] *Sixuan Duan,*[1, 2, 5] *Shan Cong,*[*, 6] *Ruiqi Yong,*[1] *Feifan Li,*[6] *and Pengfei Song*[*, 1,2]

#These authors contributed equally to this work and should be considered co-first authors.

1 School of Advanced Technology, Xi'an Jiaotong - Liverpool University, 215123 Suzhou, China

2 Department of Electrical Engineering and Electronics, University of Liverpool, L69 7ZX Liverpool, UK

3 State Key Laboratory for Manufacturing Systems Engineering, Xi'an Jiaotong University, 710049 Xi'an, China

4 School of Physical Science and Technology, Suzhou University of Science and Technology, 215009 Suzhou, China

5 Key Laboratory of Bionic Engineering, Jilin University, 130022 Changchun, China

6 School of Nano-Tech and Nano-Bionics, University of Science and Technology of China, 215123 Suzhou, China

[*] Pengfei Song; Phone: +86 (0)512-81889039; Pengfei.Song@xjtlu.edu.cn

[*] Shan Cong; Phone: +86 (0)512-62872552; scong2012@sinano.ac.cn

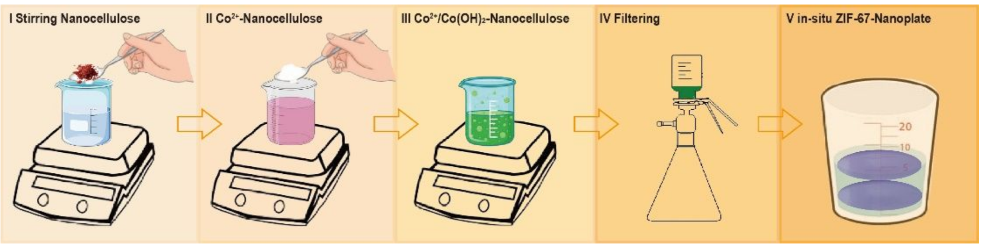

Figure S1. Schematic diagram of *in-situ* ZIF-67 Nanoplate fabrication process.

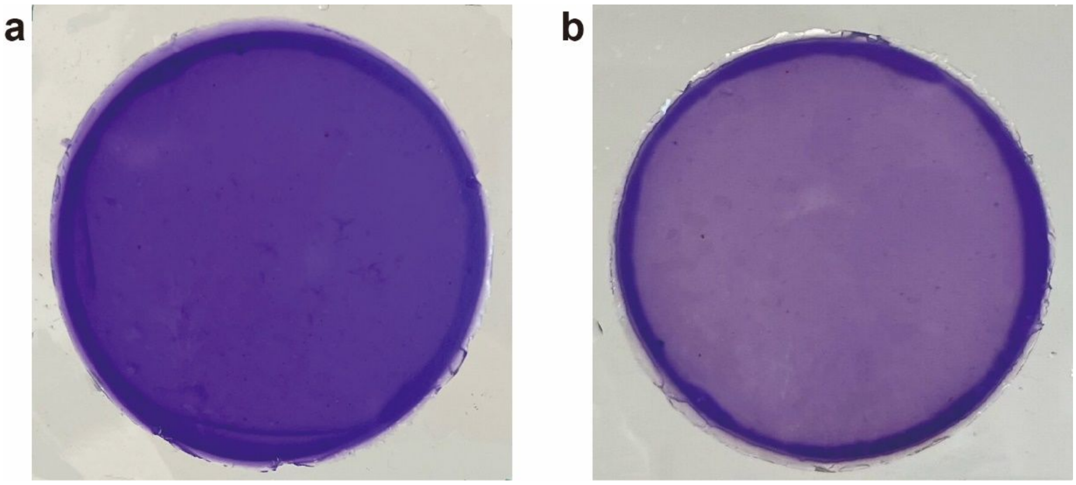

Figure S2. Photographs of *in-situ* ZIF-67 Nanoplate with (a) and without (b) $Co(OH)_2$ heterojunction.

Table S1. Coefficients of variation of R6G SERS detection at different concentrations.

Concentration (nM)	0	10^0	10^1	10^2	10^3	10^4	10^5
Coefficients of variation (%)	4.9	9.3	8.5	4.1	15.4	16.2	6.9

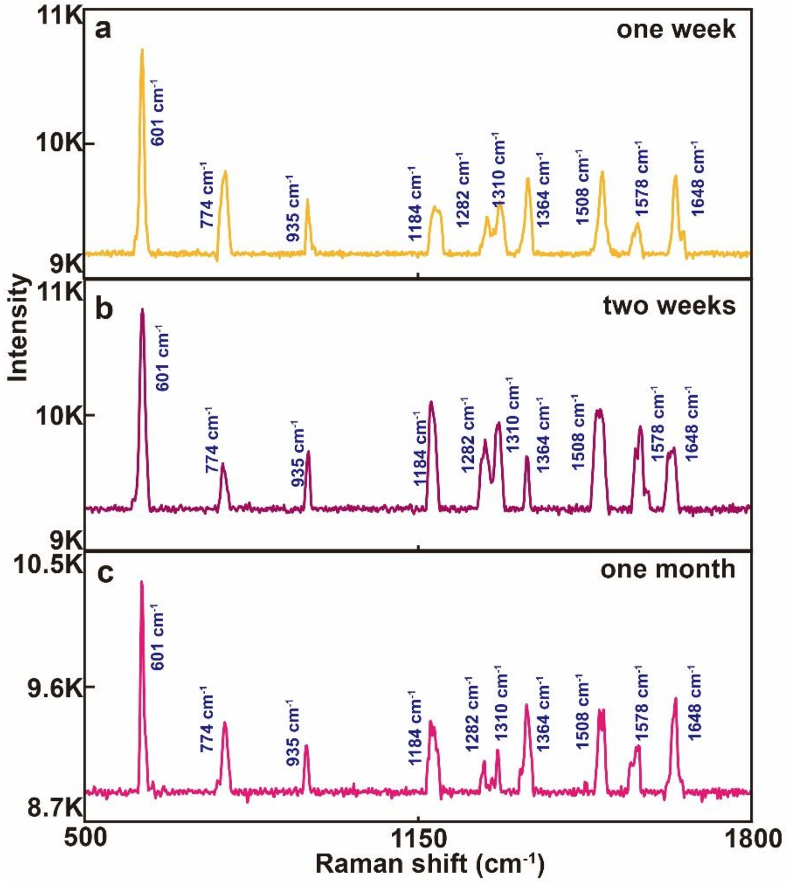

Figure S3. SERS spectra of R6G on *in-situ* ZIF-67 nanoplate storing for (a) one week, (b) two weeks and (c) one month.

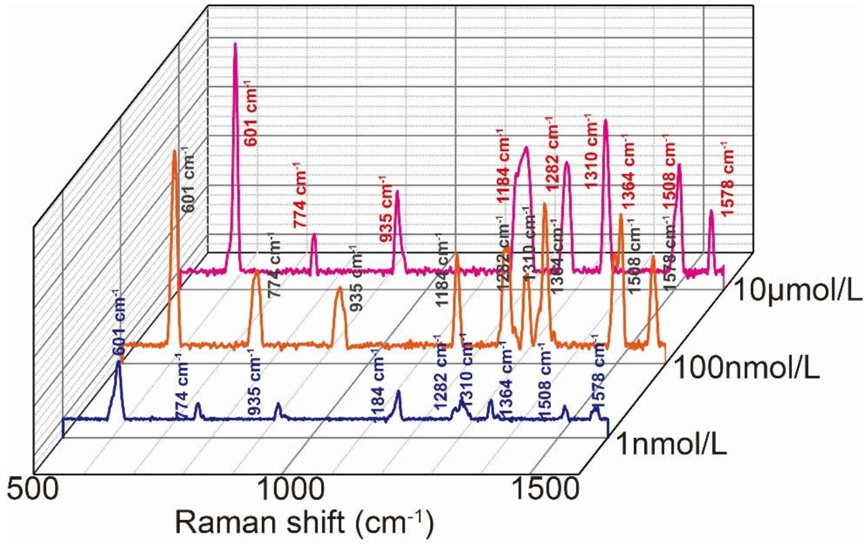

Figure S4. Specific SERS detection of R6G and RhB solution on *in-situ* ZIF-67 nanoplate, and only R6G's characteristic peak was detected.

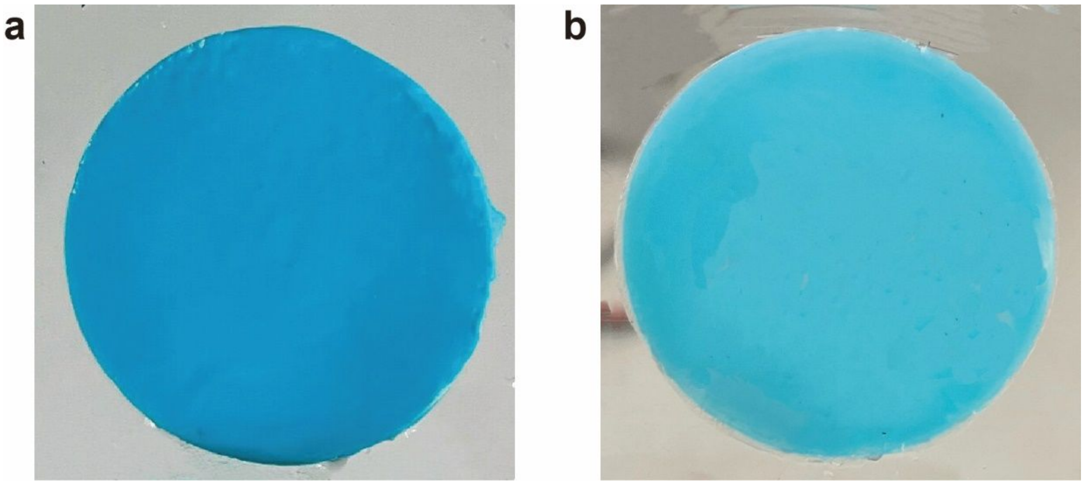

Figure S5. Photographs of *in-situ* HKUST-1 Nanoplate with (a) and without (b) $Cu(OH)_2$ heterojunction.

Table S2. Coefficients of variation of BTC SERS detection at different concentrations.

Concentration (nM)	0	10^{-3}	10^{-4}	10^{-5}	10^{-6}	10^{-7}	10^{-8}
Coefficients of variation (%)	19.0%	9.6%	7.1%	5.2%	8.4%	11.0%	9.9%

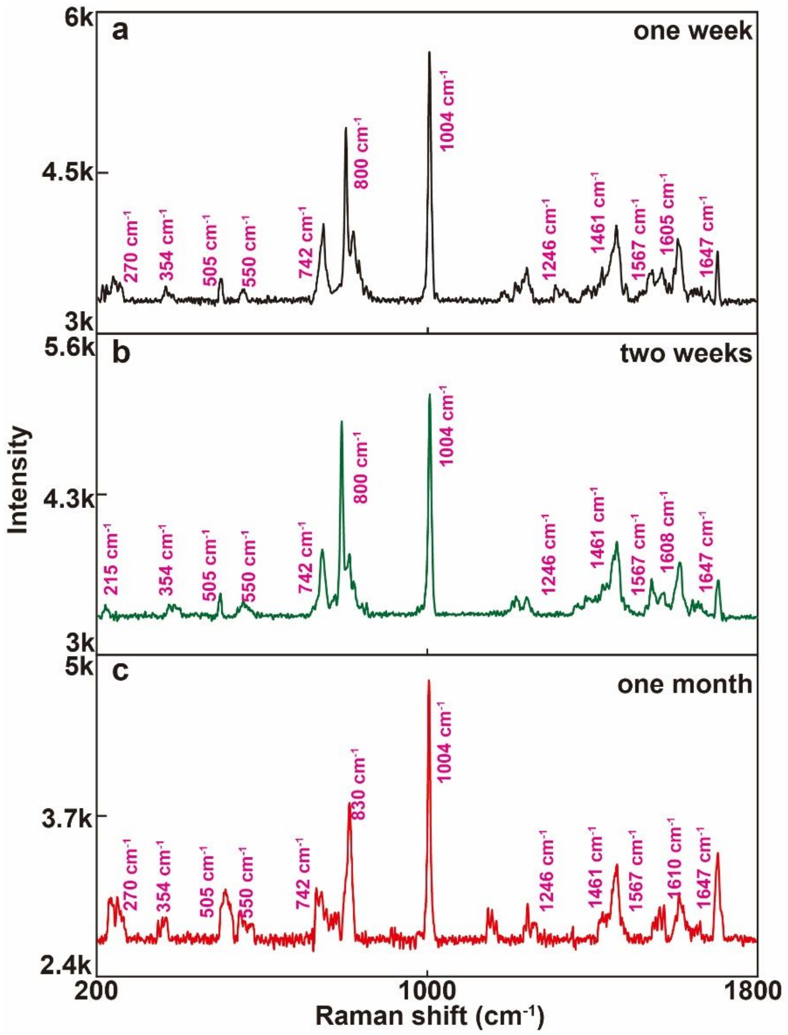

Figure S6. SERS spectra of BTC on *in-situ* HKUST-1 nanoplate storing for (a) one week, (b) two weeks and (c) one month.
